# Assessing cardiovascular parameters and risk factors in physical therapy practice: findings from a cross-sectional national survey and implication for clinical practice

**DOI:** 10.1186/s12891-022-05696-w

**Published:** 2022-08-04

**Authors:** Agostino Faletra, Giuseppe Bellin, James Dunning, César Fernández-de-las-Peñas, Leonardo Pellicciari, Fabrizio Brindisino, Erasmo Galeno, Giacomo Rossettini, Filippo Maselli, Richard Severin, Firas Mourad

**Affiliations:** 1 Clinical Support & Screening Service, Queen Elizabeth Hospital, Gateshead, United Kingdom; 2 Department of Physical Therapy, Centro Diagnostico Veneto, Vicenza, Italy; 3 American Academy of Manipulative Therapy Fellowship in Orthopaedic Manual Physical Therapy, Montgomery, Alabama USA; 4 Montgomery Osteopractic Physiotherapy & Acupuncture Clinic, Montgomery, Alabama USA; 5 Department of Physical Therapy, Occupational Therapy, Rehabilitation and Physical Medicine, Universidad Rey Juan Carlos, Alcorcón, Spain; 6 Cátedra de Investigación, Clínica y Docencia en Fisioterapia: Terapia Manual, Punción Seca y Ejercicio, Universidad Rey Juan Carlos, Madrid, Alcorcón Spain; 7 IRCCS Istituto delle Scienze Neurologiche di Bologna, Bologna, Italy; 8 Department of Medicine and Health Science “Vincenzo Tiberio”, University of Molise c/o Cardarelli Hospital, Campobasso, Italy; 9 Department of clinical science and translation medicine, University of Rome Tor Vergata, Roma, Italy; 10 Polimedico Specialistico STEMA Fisiolab, Latina, Italy; 11 Department of Medical Sciences, surgery and neuroscience, Università degli studi di Siena, Siena, Italy; 12 Department of Human Neurosciences, “Sapienza” University of Rome, Rome, Italy; 13 School of Physiotherapy, University of Verona, Verona, Italy; 14 Sovrintendenza Sanitaria Regionale Puglia INAIL, Bari, Italy; 15 Department of Physical Therapy, University of Illinois at Chicago, College of Applied Health Sciences, Chicago, IL USA; 16 Department of Physical Therapy, Baylor University, Robbins College of Applied Health Sciences, Waco, TX USA; 17 Department of Physiotherapy, LUNEX International University of Health, Exercise and Sports, 4671 Differdange, Luxembourg; 18 Luxembourg Health & Sport Sciences Research Institute, A.s.b.l., 50, Avenue du Parc des Sports, 4671 Differdange, Luxembourg

**Keywords:** blood pressure, heart rate, cardiovascular disease, prevention, differential diagnosis, cardiovascular risk factor

## Abstract

**Background:**

Cardiovascular diseases are the leading cause of death and comorbidity worldwide. High blood pressure and resting heart rate are risk factors (or vital signs) critical to cardiovascular health, patient safety, and medical management. Physiotherapists play a fundamental role in risk factor identification, early diagnosis, and subsequent management of cardiovascular disease. To date there is limited research in Europe investigating the level of knowledge and skills possessed by physiotherapists regarding cardiovascular disease screening. Three studies previously observed inadequate vital signs screening behaviors of physiotherapists practicing in the United States and Saudi Arabia. The primary aim of this study was to investigate cardiovascular knowledge and screening practices among Italian physiotherapists, according to the current practice recommendations.

**Methods:**

A Cross-Sectional Survey was developed adapting two previous surveys. The survey was administered to members of the Italian Physiotherapy Association. Chi squared test, Mann-Whitney test or Kruskal-Wallis test were used to study differences among subgroups and question responses.

**Results:**

The required sample size was met with total of 387 Italian physiotherapists completing the survey. 80% consider relevant cardiovascular assessment. However, 72.2% were not familiar to guidelines recommendations and only 50% screen vital signs routinely. Their knowledge of normative blood pressure (high-normal, 16%; hypertension, 12%) and heart rate values (bradycardia, 24%; tachycardia, 26%) were low. Although participants reported being skilled for blood pressure measurement (quite sure, 52%; sure, 27%), their adherence to guidelines is low (baseline measurement on both arm, 25%; 3 repeated measures, 46%). Only 27.8% reported to measure exercise related BP and 21.3% of them understood the concept of exaggerated BP. No significant differences between subgroups were found.

**Conclusions:**

Our study revealed that a concerning proportion of Italian physiotherapists are not versed in fundamentals of properly performing cardiovascular screenings. This lack of knowledge is present across the profession and may impact on appropriate triage and management. The poorly executed screening has the potential to negatively impact the patient and the practitioner. Given the absence of Italian guidelines, we produced and implemented three infographics for public use, which have the dual objective of raising awareness about this subject and providing practical resources for everyday practice.

**Supplementary Information:**

The online version contains supplementary material available at 10.1186/s12891-022-05696-w.

## Background

Patients with musculoskeletal disorders frequently present with significant cardiovascular risk factors and disease (CVD). Patients may also present with unrecognized or undiagnosed risk factors and disease, which may require referral [1–5]. An estimated 69% of outpatient musculoskeletal physical therapists encounter patients daily if not twice per week with an established diagnosis of or moderate/high risk factors for cardiovascular disease [2]. Early detection and timely referral of unrecognized CVD ensures patient safety and appropriate medical management [6, 7]. Physiotherapists, particularly with direct access, must routinely screen for blood pressure (BP) and heart rate (HR) to identify risk factors, support recommended management, and ensure medical referrals [1, 8–13].

CVD arises from an interplay between a multitude of factors and is the leading cause of disability-adjusted life years worldwide, morbidity and mortality [14–17]. High blood pressure (HBP) is the leading risk factor for CVD among the most common risk factors (i.e., smoking, diabetes, dyslipidemia/hypercholesterolemia, obesity, physical inactivity/low fitness, unhealthy diet, family history, male sex, obstructive sleep apnea, psychological stress) [18]. The prevalence of HBP in central and eastern Europe is estimated to be over 150 million, reaching 30-45% in adulthood, with a peak of 60% in ages over 60 years old [19].

Recent estimations suggest a positive correlation between increased systolic (SBP) and diastolic blood pressure (DBP) and CVD, i.e. the higher the pressure, the greater the risk [6]. For prevention and treatment purposes, the most recent guidelines developed by the International and the European society of Hypertension (ISH and ESH) recommended to categorize HBP as normal, elevated, or stage 1 or 2 hypertension. Based on a careful reading obtained on the average of ≥2 occasions, after having selected the arm with the higher reading, normal blood pressure for most adults is defined as a SBP of <130mmHg and a DBP of <85mmHg. HBP should be categorized as high-normal (130–139mmHg and/or <85-89mmHg), hypertension grade 1 (140–159mmHg and/or 90–99 mm Hg), and hypertension grade 2 (≥160mmHg and/or ≥100mmHg) [20]. Notably, when SBP and DBP measurements are recorded in 2 different categories, the individual should be designated to the higher one [18]. A report suggests over 55% of patients are unaware of having HBP due to its often asymptomatic nature which may contribute to an increased risk of mortality [6]. Furthermore, masked hypertension (MH) is a common event (14-30% in normotensive population) which occurs when a hypertensive patient presents with a normal BP reading [18, 21]. It was estimated that an exaggerated BP response to exercise and physical activity could detect 41% of MH [17, 22, 23]. Exercise BP response is unscreened by both medical doctors and physiotherapists although the American Heart Association (AHA) strongly recommends MH detection, [2, 18]. Exaggerated BP increase to exercise has also been found to be a valuable screening and prognostic tool for both unmanifested CVD and future cardiovascular events [24]. The AHA defined exaggerated increases in BP in response to exercise as SBP >210mmHg for men and >190mmHg for women, and a DBP increase >10mmHg above resting values [25].

Heart rate (HR) is a parameter/vital sign important to those patients at risk of CVD. An elevated resting heart rate increases the likelihood of future cardiovascular pathologies and becomes particularly significant above 90 beats per minute [26]. HR is an indirect parameter of myocardial oxygen demand, coronary blood flow and myocardial performance that can be easily and non-invasively measured. Elevated resting heart rate in combination with HBP is associated with a further increased risk of mortality [26].

To date there is limited research investigating the level of cardiovascular screening knowledge and psychomotor skills possessed by practicing physiotherapists. Two studies previously observed inadequate screening behaviors of physiotherapists in the United States and Saudi Arabia towards BP and HR assessment observing inadequate screening behaviors [2, 27]. However, this question has not been investigated in Europe. In Italy cardiovascular rehabilitation is included during the entry level training for physiotherapists. Italian physiotherapists are also increasingly practicing as direct access providers, which potentially makes them the first point of contact within the healthcare system [28–30]. Therefore, it is imperative that Italian physiotherapists understand the importance of CVD screening, and demonstrate competency with examination techniques in order to safely manage these cases, and confidently communicates the need for referral when necessary [31]. Therefore, the purposes of our study were to 1) investigate the knowledge and the utilization of cardiovascular screening among Italian physiotherapists and 2) to develop a resource to improve clinical practice for cardiovascular screening in the physiotherapy outpatient setting.

## Methods

We developed an online cross-sectional survey using the online platform Survey Monkey (SVMK Inc., San Mateo, USA) addressed to Italian physiotherapists. The investigation was designed in line with the Checklist for Reporting Results of Internet E-Surveys [32] and the Strengthening the Reporting of Observational studies in Epidemiology guidelines [33]. The sample was comprised of physiotherapists practicing in Italy, all other healthcare professionals were excluded.

### Survey development

We adapted two previous published surveys (with permission from the authors) to develop our version for the Italian population [2, 34]. Given the different Italian socio-legal and cultural context, based on the ISH and ESH guidelines [20, 35] and the clinical recommendations by Severin et al. [1], we adapted the questionnaire on current recommendations on CVD screening by physiotherapists to investigate the Italian physiotherapists knowledge of cardiovascular assessment and interpretation of its results. The questionnaire was revised by two authors (native English and Italian, and specialized orthopedic manipulative physical therapy—OMPT--) with experience in education and research (FM and AF). Finally, it was piloted by five experienced Italian, American and Spanish physiotherapists and one physician (EG, CF, JD, GB, RS, and MG) for additional feedback on procedural and logical accuracy and fulfillment duration. The combined use of previous surveys and expert opinion provided during the piloting process strengthened the content and face validity of the survey [2, 34].

The questionnaire was divided into three sections. The first section investigated demographics, practice settings and education level. The second addressed the knowledge of current practice recommendations, cardiovascular parameters, risk factors, blood pressure measurement technique and clinical management. The last one focused on formal education and personal opinions. The survey consisted of 32 questions using a combination of close-ended (few of them with multiple selection) and Likert-scale (Additional file 1). All questions were presented in the same order and full completion was mandatory to be considered eligible.

### Setting and recruitment

The web-link to the survey was distributed via mailing list of the Italian Physiotherapists Association on 23^rd^ of March 2020. To take advantage of the forced lock-down period due to the Covid-19 pandemic and to maximize the response rate, invitations to participate were frequently re-published one per week via social media networks (Facebook, Twitter, LinkedIn, and Instagram). The survey was open for one month and the closing date was the 26^th^ of April 2020. Consistent with previous similar survey studies, we adopted this methodological approach with the aim to collect the maximum number of answers within a given period as the majority of responses tend to occur early after posting [36–38]. A priori, a sample size was calculated using the e-survey Dillman’s formula with a 95% confidence level and a 5% of margin of error. At the time of the survey, the number of physiotherapists registered to the association was 7398; therefore, the required sample size for this study was 366 [39].

After reviewing the introduction (background, aims and content) participants had the opportunity to ask any questions and to provide their consent to proceed. The questionnaire could be completed on any electronic device with internet access; as Survey Monkey was used without collecting respondents’ IP addresses. The recruitment was anonymous and voluntary, but the same IP was not allowed to access to the survey more than once. The completion took approximately 10-15 minutes. No compensation or reimbursement were offered. Participants were able to provide as much information as they prefer and could stop completion of the survey at any point.

### Data processing and statistical analysis

There was no hard copy of the data and no sensitive information were collected. All data were organized and analyzed in Microsoft Excel 2020. Categorical data were reported as frequencies. All graphs and tables were created using the same software. Descriptive statistics were used to describe the sample; especially, absolute frequency with its percentage was calculated for each categorical variable and mean±standard deviation (SD) was computed for answers to Likert-question.

In order to study differences among sample subgroups (OMPT qualification, patients access regimen, and years of practice) and the response to the categorical questions, a Chi squared was performed. When significant differences were detected (*p*<0.05), adjusted standardized residuals with Bonferroni-corrected *p*-values were calculated in order to identify which cells of contingency table contributed most to the significant effect [40, 41].

As the Kolmogorov-Smirnov test showed a non-normal data distribution, a Mann-Whitney test or a Kruskal-Wallis test with Bonferroni-corrected post-hoc comparisons was run for two-categories (i.e., OMPT qualification, access regimen) or five-categories subsample (i.e., years of practice), respectively, to study any difference between sample subgroups and the response to the ordinal (i.e., Likert) questions.

All statistical analyses were performed with SPSS software (SPSS. Version 20 for Windows; SPSS Inc., Chicago, IL, USA, 2004), and the level was set at *p*-value < 0.05 for all comparisons.

## Results

### Responses

Of the 764 different IP addresses that provided consent and accessed the survey, 387 completed the questionnaire (5.2%, *n*=387/7398). Although available for a very short period, our sample was in line with previous Italian surveys, and reached the required sample size [42–44].

### Respondent characteristics

Around half of the participants (56%, *n*=220; 95%CI 51.6-61.5) possessed only bachelor degree; only 35% (*n*=139; 95%CI 31.0–40.5) had an OMPT qualification [38]; nearly two thirds worked in private practice (66%; *n*=258; 95%CI 61.6–71.0); and about half (57%, *n*=223; 95%CI 52.4-62.2) primarily as direct access practitioner. Twenty-seven percent (*n*=106; 95%CI 22.8–31.7) of respondents had been practicing for 5 to 10 years. Further demographic data are reported in Table 1.

**Table 1:** Section 1 survey responses

Section 1. Demographics, practice settings and the education level characteristics			
	**N**	**%**	**95 CI**
**What is your highest earned degree?**			
BSc	220	56.6	51.6-61.5
MSc	163	41.9	37.0-46.8
**Did you earn an IFOMPT OMPT specialization?**			
Yes	139	35.7	31.0-40.5
No	248	63.8	59.0-68.5
**How many years have you been practicing as a licensed physical therapist?**			
0-5	89	22.9	18.7-27.1
6-10	106	27.2	22.8-31.7
11-15	70	18.0	14.2-21.8
16-20	43	11.1	7.9-14.2
20+	79	20.3	16.3-24.3
**What physical therapy setting(s) do you currently practice in?** ^a^			
Private practice (primary line care)	258	66.3	61.6-71.0
Hospital (secondary care line)	192	49.4	44.4-54.3
Education	22	5.7	3.4-8.0
Research	9	2.3	0.8-3.8
**What main physical therapy access regimen do you practice in?**			
Direct access	223	57.3	52.4-62.2
Secondary care referral pathway	163	41.9	37.0-46.8

*Abbreviations***.** *IFOMPT* International Federation of Orthopaedic Manipulative Physical Therapists, *OMPT* Orthopaedic Manipulative Physical Therapist

^a^select ALL that apply

Table 2 provide the responses of section 2 and 3 of the survey.

**Table 2:** Section 2 and 3 survey responses

**Section 2. Knowledge of current practice recommendations, cardiovascular parameters, risk factors, blood pressure measurement technique and clinical management**			
**Are you familiar with guidelines relevant to cardiovascular parameters assessment?**	**N**	**%**	**95 CI**
Yes	106	27.2	22.8-31.7
No	281	72.2	67.8-76.7
**Which international guidelines are you familiar with**^a^			
NICE	43	40.6	31.2-49.9
American Heart Association	58	54.7	45.2-64.2
Italian Society of Hypertension	26	25.5	16.3-32.7
European Society of Cardiology and European Society of Hypertension	40	37.7	28.5-47.0
**Do you consider cardiovascular assessment in your clinical practice?**			
Yes	311	79.9	76.0-83.9
No	76	19.5	15.6-23.5
**If not, why you don’t evaluate the cardiovascular parameters?**			
Not relevant for my practice	56	73.7	63.8-83.6
Outside the physical therapy’s scope	6	7.9	1.8-14.0
Work in secondary care referral pathway	14	18.4	9.7-27.1
**If yes, what cardiovascular parameter do you evaluate?** ^a^			
Heart rate	249	80.1	75.6-84.5
Blood pressure	266	85.5	81.6-89.4
Oxygen saturation	184	59.2	53.7-64.6
None	4	1.3	0.0-2.5
**If yes, what are the most relevant risk factors?** ^a^			
Smoke	266	85.5	81.6-89.4
Diet	151	48.6	43.0-54.1
Level of fitness	146	46.9	41.4-52.5
Cholesterol	165	53.1	47.5-58.6
Diabetes	168	54.0	48.5-59.6
Obesity	241	77.5	72.9-82.1
Chronic kidney disease	39	12.5	8.9-16.2
Familiarity	140	45.0	39.5-50.5
Old age	42	13.5	9.7-17.3
Socioeconomic level	10	3.2	1.3-5.2
Gender	10	3.2	1.3-5.2
Sleep apnea	37	11.9	8.3-15.5
Stress	80	25.7	20.9-30.6
**Which are the normal, high-normal blood pressure ranges (systolic blood pressure; diastolic blood pressure) defined in the most recent guidelines?**			
Normal (SBP<130/DBP<85)	260	66.8	62.2-71.5
High-normal (SBP =130-139/DBP=85-89)	64	16.5	12.8-20.1
Hypertension (SBP >140/DBP>90)	47	12.1	8.8-15.3
**Which are the normative values of the heart rate defined in the most recent guidelines?**			
Normal (HR=60-100 bpm)	305	78.4	74.3-82.5
Bradycardia (HR<60bpm)	95	24.4	20.2-28.7
Tachycardia (HR>100bpm)	102	26.2	21.9-30.6
**Have you ever received an adequate training, or have you ever done any specific courses on the measurement of the cardiovascular parameters?**			
Yes	121	31.1	26.5-35.7
No	266	68.4	63.8-73.0
**If yes, where did you learn these notions?** ^a^			
Workplace	45	37.2	28.6-45.8
Continuing Professional Development courses	42	34.7	26.2-43.2
During the Bachelor	91	75.2	67.5-82.9
During the Master	14	11.6	5.9-17.3
Interaction with other healthcare professionals	50	41.3	32.5-50.1
Personal readings (scientific books or literature)	27	22.3	14.9-29.7
Social media and Podcast	14	11.6	5.9-17.3
**How relevant is cardiovascular parameters assessment in your practice?**	**Mean**	**SD**	
Likert (0-10)	7.4	2.1	
**Do you measure blood pressure and/or heart rate in your practice?**	**N**	**%**	**95 CI**
Yes	196	50.4	45.4-55.4
No	186	47.8	42.9-52.8
**If no, why?**			
Outside the physical therapy scope of practice	112	60.2	53.2-67.2
Working in a secondary care referral pathway (patients previously evaluated by a physician)	73	39.2	32.2-46.3
Not trained adequately	43	23.1	17.1-29.2
Requires too much time	20	10.8	6.3-15.2
**Quantify your ability in conducting a blood pressure assessment**			
Not confident	5	1.3	0.2-2.4
Insecure	73	18.8	14.9-22.6
Quite sure	204	52.4	47.5-57.4
Sure	104	26.7	22.3-31.1
**Quantify your confidence in interpreting the findings within your blood pressure assessment**			
Not confident	11	2.8	1.2-4.5
Insecure	104	26.7	22.3-31.1
Quite sure	213	54.8	49.8-59.7
Sure	55	14.1	10.7-17.6
**Quantify your confidence in managing the findings within your blood pressure assessment disorders**			
Not confident	14	3.6	1.7-5.4
Insecure	139	35.7	31.0-40.5
Quite sure	188	48.3	43.4-53.3
Sure	41	10.5	7.5-13.6
**Quantify your ability in conducting a heart rate assessment**			
Not confident	6	1.5	0.3-2.8
Insecure	64	16.5	12.8-20.1
Quite sure	199	51.2	46.2-56.1
Sure	116	29.8	25.3-34.4
**Quantify your confidence in interpreting the findings within your heart rate assessment**			
Not confident	11	2.8	1.2-4.5
Insecure	115	29.6	25.0-34.1
Quite sure	189	48.6	43.6-53.6
Sure	70	18.0	14.2-21.8
**Quantify your confidence in managing the findings within your heart rate assessment**			
Not confident	14	3.6	1.7-5.4
Insecure	147	37.8	33.0-42.6
Quite sure	166	42.7	37.8-47.6
Sure	58	14.9	11.4-18.4
**How many baseline blood pressure measurements are recommended?**			
Other (<3, >3)	194	49.9	44.9-54.8
3	181	46.5	41.6-51.5
**Where should the blood pressure measurement have to be performed?**			
Other (left arm, right arm, no difference)	288	74.0	69.7-78.4
Both	97	24.9	20.6-29.2
**If both, which indications should be given to the patient?**			
Other (Monitor both arms, Average the measurements of the two arms, No specific indication)	52	53.6	43.7-63.5
Monitor the arm with the highest blood pressure	45	46.4	36.5-56.3
**How would you manage any serious anomalies detected during the evaluation of cardiovascular parameters?** ^a^			
Monitoring patient’s symptoms	258	66.3	61.6-71.0
Refer to general practitioner	203	52.2	47.2-57.1
Referral to the emergency department	166	42.7	37.8-47.6
Referral to a specialist	128	32.9	28.2-37.6
Request further examination	61	15.7	12.1-19.3
**To what extent do you consider cardiovascular risk in your practice?**	**Mean**	**SD**	
Likert (0-10)	4.2	2.3	
**Do you screen for cardiovascular risk in your practice?**	**N**	**%**	**95 CI**
Yes	273	70.2	65.6-74.7
No	112	28.8	24.3-33.3
**Do you evaluate your patients’ cardiovascular fitness before exercises?**			
Yes	130	33.4	28.7-38.1
No	256	65.8	61.1-70.5
**If yes, what tools or tests do you use to assess the cardiovascular fitness of your patients?** ^a^			
Maximal incremental test	12	9.2	4.3-14.2
6 minutes walking test	86	66.2	18.0-26.2
3 minutes step up test	25	19.2	12.5-26.0
Indirect tests	48	36.9	28.6-45.2
None	4	3.1	0.1-6.0
**Do you monitor blood pressure values prior, during and/or post-exercise?**			
Yes	108	27.8	23.3-32.2
No	277	71.2	66.7-75.7
**If yes, please specify the current recommended upper blood pressure threshold (systolic blood pressure; diastolic blood pressure) triggering the cessation of exercise.**			
Exaggerated Blood Pressure values (SBP>250/>DBP>115)	23	21.3	13.6-29.0
**Section 3. Formal education and personal opinions**			
**How much relevant do you consider training in cardiovascular parameter assessment?**	**Mean**	**SD**	
Likert (0-10)	7.1	2.3	
**How much relevant do you consider training in cardiovascular risk assessment/management (e.g., syncopal events, tachycardia etc.)?**			
Likert (0-10)	8.3	1.9	
**How training in conducting cardiovascular assessment should be provided?** ^a^	**N**	**%**	**95 CI**
Within the under-graduate programs (Bachelor)	320	82.3	78.5-86.1
Within post-graduate programs (Masters)	81	20.8	16.8-24.9
Within Continuing Professional Development courses	165	42.4	37.5-47.3
In the workplace	185	47.6	42.6-52.5

^a^select ALL that apply

### Knowledge, understanding and skills towards cardiovascular assessment

The majority of participants (72%, *n*=281; 95%CI 67.8–76.7) were not up to date with international recommendations for the assessment of cardiovascular parameters; among those who were (55%; *n*=58; 95%CI 45.2–64.2), the AHA guidelines was one known the best.

A great proportion of physiotherapists (80%; *n*=311; 95%CI 76.0–83.9) reported that they take cardiovascular parameters into account in their everyday practice; of those, 80% (*n*=249; 95%CI 75.6–84.5) reported measuring the HR and 86% (*n*=266; 95%CI 81.6–89.4) the BP. For those who did not consider them (20%; *n*=76; 95%CI 15.6–23.5), the most common reason was “not relevant for clinical practice” (73%; *n*=56; 95%CI 63.8–83.6). Smoking (86%; *n*=266; 95%CI 81.6–89.4) and obesity (78%; *n*=241; 95%CI 72.9–82.1) were the most reported risk factors. When compared against the ISH guidelines, their knowledge of normative BP values is respectively 66% (*n*=260; 95%CI 62.2–71.5) for normal range, 16% (*n*=64; 95%CI 12.8–20.1) for high-normal BP, and 12% (*n*=47; 95%CI 8.8–15.3) for hypertension. Note that hypertension was asked as a grouped item (i.e., not as 2 separate grades). Interestingly, 78% (*n*=305; 95%CI 74.3–82.5) of the respondents were aware of the HR normal value; however only 24% (*n*=95; 95%CI 20.2–28.7) and 26% (*n*=102; 95%CI 21.9–30.6) responded adequately respectively for bradycardia and tachycardia values. Only 31% (*n*=121; 95%CI 26.5–35.7) admitted having received adequate training in appraising cardiovascular parameters, and the majority were trained during their bachelor degree course (75%, *n*=91; 95%CI 67.5–82.9).

Although a moderate number considered cardiovascular parameters assessment relevant for their routine practice (mean=7.4/10 points; SD=2.1), only 50% (*n*=196; 95% CI 45.4 – 55.4) routinely measure BP and/or HR. Being outside physiotherapists’ scope of practice was the leading justification reported for not performing these assessments (60%, *n*=112; 95% CI 53.2 – 67.2).

The participants reported being skilled for both BP (“quite sure” and “sure” respectively, 52%; *n*=204; 95%CI 47.5–57.4, and 27%; *n*=104; 95%CI 22.3–31.1) and HR measurements (“quite sure” and “sure” respectively, 51%; *n*=199; 95%CI 46.2–56.1, and 30%; *n*=116; 95%CI 25.3–34.4). The majority reported being “quite sure” in interpreting and managing BP measurement (respectively, 55%; *n*=213; 95%CI 49.8–59.7, and 48%; *n*=188; 95%CI 43.4–53.3) and HR measurement (respectively, 48%; *n*=189; 95%CI 43.6–53.6, and 42%; *n*=166; 95%CI 37.8–47.6). Only 25% (*n*=97; 95%CI 20.6–29.2) measured BP on both arms at the initial examination, and only 46% (*n*=181; 95%CI 41.6–51.5) of them reported that they repeat measurements three times as recommended. Notably, of those that measure on both arms, only 46% (*n*=45; 95%CI 36.5–56.3) reported that they continued assessing the limb with the highest BP for future screening.

Physiotherapists’ opinion towards the relevance of the assessment for cardiovascular risk factors was scored at 4.2/10 (SD=2.3). Of the respondents, 70% (*n*=273; 95%CI 65.6–74.7) screened for cardiovascular risk in clinical practice, but only 33% (*n*=130; 95%CI 28.7–38.1) assessed their patients’ cardiovascular fitness before exercising. When this assessment is performed the tool reported being used the most is the 6 minutes walking test (66%; *n*=86; 95%CI 18.0–26.2). Furthermore, only 28% (*n*=108; 95%CI 23.3–32.2) declared to monitor BP during and/or post-exercise, and of them only 21% (*n*=23; 95%CI 13.6–29.0) acknowledged the most accepted values of exaggerated increases in BP in response to exercise.

### Cardiovascular assessment training

Our participants considered that training in cardiovascular assessment was highly relevant for physiotherapists (mean 8.3/10; SD=1.9). Most of them (82%; *n*=320; 95%CI 78.5–86.1) reported that training in cardiovascular assessment should be provided during a physiotherapy degree as part of the core curriculum.

### Differences between sample subgroups

The non-parametric tests did not reveal any significant differences (*p*>0.05) for the subgroup samples (i.e., highest earned degree, OMPT qualification, access regimen, and years of practice) (Additional file 2).

## Discussion

### Key findings

This is the first study which provides insights from an Italian physiotherapists cohort about their knowledge, understanding and screening behaviors in the assessment of cardiovascular parameters and risks factors. Our results highlight that cardiovascular assessment is considered relevant by 80% of physiotherapists. Fifty percent of the respondents reported that they routinely include cardiovascular assessment in their initial examination. However, given the number of professionals who work in a first contact role, it is concerning that 72.2% were not familiar to current practice recommendations and a substantial proportion were not familiar with the fundamentals of cardiovascular measurements when compared against the ISH guidelines. Clinical Practice Guidelines are paramount to implement the most updated available knowledge in clinical practice [45, 46]. Additionally, as the Italian Physiotherapy Association advocates for direct access [28–30], Clinical Practice Guidelines knowledge is a professional responsibility [47]. Surprisingly, 60.2% reported that cardiovascular measurements are outside their scope of practice. This finding is similar across the profession as indicated by previous studies conducted in other countries [1, 10, 34, 48]. For example, although the American Physical Therapist Association Guide to Physical Therapist Practice encourages early screening for medical conditions, including a review of the cardio-vascular/pulmonary system by measuring HR and BP [8], it was observed that only 10-15% of U.S. outpatient physiotherapists did perform a routine cardiovascular assessment because it is believed to be beyond their competence [1]. Similar findings were found in Saudi Arabia [27]. Our results highlight a large discrepancy between the number of physiotherapists that consider cardiovascular assessment and that actually do it. Even more importantly, there is a large discrepancy between those that consider cardiovascular assessment and those skilled to measure it and that understand what it means. These barriers seem to result from gaps in knowledge and policy. Changing the perception of the importance of cardiovascular assessment can be addressed through education and knowledge translation of both clinicians and students [2, 49]. Also, Institutional or professional polices can be developed to require cardiovascular assessment, thus helping to include cardiovascular assessment in clinical policies and standards [2]. Furthermore, the development of clinical practice guidelines specifically directed to physiotherapists could assist with this process by making the clinical understanding and application of cardiovascular assessment better understood in order to follow through with appropriate recommendations for education, exercise, and referrals.

Notably, most of participants from the current cohort felt confident in conducting and interpreting cardiovascular measurements. However, a very small proportion were updated on how to perform a thorough BP assessment (i.e., the average of three measurements of the arm with the highest blood pressure) and on the knowledge of the BP and HR normative values [20, 35]. The absence of significant differences between groups (e.g., possessing a specialization or working in a direct access setting) suggests that this inadequacy is homogeneously distributed across the profession. This may reflect the absence of Italian recommendations for the best practice on the subject.

As physiotherapy practice advances to a more independent care model [31], the screening for cardiovascular risk factors and related serious pathologies become of paramount importance to ensure patient safety and effective medical management [20]. This becomes even more relevant in light of the alarming number of CVD-related deaths [50]. Given that hypertension is frequently asymptomatic, even at critical high values (SBP 180 mm/Hg - DBP 120 mm/Hg), its early detection is fundamental for a prompt medical treatment and associated reduction of mortality and morbidity [7]. Physiotherapists, as first contact health-care professionals, especially when working in a direct access setting, could ensure timely medical referral for further investigations through an accurate screening [1].

Furthermore, commonly treated musculoskeletal presentations such as neck pain, could potentially hide more serious pathologies related to the cardiovascular system [51–53]. That is, HBP and elevated HR are relevant in the pathogenesis of either an aneurysm or a vascular stenosis that underlie a spontaneous cervical artery dissection in older adults, and are important predictors to inform for any acute damage/trauma of cervical arteries in younger population [54, 55]. Also, It has been reported that some patients with neck disorders who underwent cervical traction may experience a significant drop in their HR and BP resulting from temporary alteration of the autonomic nervous system processes [56]. These temporary responses to very commonly utilized musculoskeletal treatments, in highly predisposed subjects (e.g., elderly patients or patients undertaking medications, such as non-steroidal anti-inflammatory or corticosteroids drugs), may increase the risk of falling, which could result in serious and potentially fatal complications. This emphasizes the importance of physiotherapists possessing strong knowledge of the cardiovascular system and routinely implementing CVD assessment during their daily practice irrespective of the work setting. Patients can be seen by a physiotherapist for a wide range of different disorders—back pain, knee pain, mobility issues, etc.— while, in the meantime, being managed by medical doctors for other comorbidities [57, 58]. Sixty-two percent of patients who attended an orthopedic or a direct access physiotherapy consultation also had a clinical history of cardiovascular disease [4]. Twenty-one percent of U.S. adults aged >35 years with a diagnosis of osteoarthritis also have hypertension, smoking habit and diabetes with a prevalence of 40%, 20% and 11% respectively [59]. The belief that cardiovascular screening does not apply to all physiotherapists does not truly reflect the role of a profession, which plays a pivotal role in both primary and secondary care in promoting an healthy lifestyle especially when battling physical inactivity [11, 60].

Physical inactivity alongside increasing time of sedentary behavior can have, in isolation or combination, a repercussion on the cardiovascular system with an increased disease incidence and deaths [48]. This tendency of sedentary behavior has been recently amplified by the recent Covid-19 pandemic [61, 62]. Alongside the advice of sitting less and being more physically active, the introduction of a light or moderate-vigorous intensity exercise routine has shown to be as an effective strategy to reduce the number of the aforementioned cardiovascular deaths and cardiometabolic risk factors [63–66]. Regular exercise does not only lower/optimize BP values [67] but it is also recommended as a first-line treatment for numerous musculoskeletal disorders and in the management of chronic pain [68–70]. Furthermore, the American College of Cardiology and the American College of Sports Medicine guidelines recommend regular exposure to physical activity for a period of up to 6 months before considering any pharmacological treatment, for the management of BP in low-risk category (10-year CVD risk <10%) adults with stage 1 hypertension (BP 130-139/80-89 mmHg) [71, 72].

Despite the benefits of physical activity being well known, a review of the cardiovascular system is essential before prescribing any exercise. Exercise induced hypertension (namely, exaggerated BP) has been shown to increase the risk of cardiovascular events by 1.4-3 times. Further, the risk of an exercise induced acute heart attack is seven times more likely compared to a sudden cardiac death at rest [73–75]. Hypertension is associated with a rise in cardiac infarcts and arrhythmias, which are the two major causes of sudden cardiac death during exercise among individuals aged >35 years [76–78]. Physiotherapists are uniquely positioned to screen exaggerated BP responses to physical activity and MH because exercise is an important part of physiotherapy treatment sessions. Although too little considered in our cohort, BP measurement during and after exercises plays a fundamental preventative role being able to unmask BP abnormalities in individuals with MH even during low intensity exercises [1, 79, 80]. Furthermore, an abnormal low BP response to exercise is prognostically associated with cardiovascular events and mortality regardless of exercise intensity [81]. A decrease in SBP ≥ 10 mmHg accompanied by evidence of ischemia during exercise is an absolute indication to stop all exercise [82]. Also, a decrease in BP during exercise or an increase >20-30 mmHg may indicate multiple underlying pathologies, such as myocardial ischemia [82]. Our results are in line with a previous survey which found that only 12% and 35% of U.S. physiotherapists reported monitoring BP, respectively, during and following exercises [2].

In our study, only 27.8% reported to measure BP prior, during, and/or post-exercise; 21.3% of them understood the concept of exaggerated BP values during or post exercise. As physiotherapists often prescribe exercise as part of a plan of care and decide on the intensity and type of exercise, it is imperative that physiotherapists monitor BP exercise response during their treatments for safety reasons. Obtaining information on the cardiovascular system status allows the physiotherapists to monitor normal and abnormal physiological responses to the exercise [83]. This type of screening would have the benefits of uncovering potential hidden risk factors, detecting already established CVD and triggering a timely onward referral. This would also allow a more tailored exercise prescription in order reduce the occurrence of adverse events and sudden deaths [1].

### Recommendations for clinical practice

This study has revealed an inadequate level of knowledge among Italian physiotherapists regarding cardiovascular assessment and screening. This discrepancy appears to be driven by the belief that it is not part of their professional role and does not influence their clinical practice and decision-making process. Given the increasing incidence of CVD in the general population, we strongly advise physiotherapists to perform a screening of the cardiovascular system for all patients. Specifically, BP and HR measurements must be routinely taken and documented. Despite the perceived importance of training on this topic by our cohort, training in this area needs to be strengthened to guarantee the correct identification of risk factors, recognition of red-flags, onward medical referral and, above all, patient safety. Our results show that Italian physiotherapists are still lacking specific training on this topic and how this affects their clinical decision-making process.

Further studies are needed to investigate whether differences in cardiovascular screening among physiotherapists exist depending on the work setting and how this may affect clinical practice when patients are affected by either musculoskeletal or other age-related degenerative diseases. More studies in other countries and/or based on different socio-legal background are also welcomed in order to allow a generalization of results.

Given the absence of Italian guidelines, we have produced and implemented in the article three infographics for public use, which have the dual objective of raising awareness among Italian physiotherapists and provides practical resources for everyday practice [84]. Two infographics explain the correct procedure for measuring the parameters both at rest and during exercises (Additional file 3 and 4). Also, an adjunctive infographic provides a decision tool for early identification of potential cardiovascular adverse events (Additional file 5).

### Strengths and limitations

This is the first published study conducted in Europe which describes the current knowledge and ability of physiotherapists to conduct cardiovascular assessment. The methodology used and the adaptation of previous online surveys to collect the opinion of Italian physiotherapists allowed us to successfully capture the perspective of the target population. Although we obtained the required sample size calculation, this study present limitation in the generalization on the results. Where the purpose of a study is to gain a general sense of a belief or attitude, a lower level of precision may be acceptable, and then a smaller sample size may be drawn [39]. The small response rate of our study could challenge our results in representing the target population and the generalisability of the results. We did not send personal invitations, and this may explain the low number of completed surveys. The survey completion could have also been influenced by the very detailed and specific questions that were employed. There is also a high potential for responder bias as those with stronger positive or negative opinion on the topic may be more likely to respond to the survey, or to give more detail. The used methodology may have potentially led to a selection bias. Additionally, similar to previous studies [2, 34], although the survey underwent a rigorous piloting and development process that involved a representative piloting group of European physiotherapists and physicians, the reliability of the survey was not quantified.

## Conclusions

Our study revealed that a concerning proportion of Italian physiotherapists, even those working as first line practitioners, are not trained to the fundamentals of and do not perform properly cardiovascular assessment. This appears to be driven by the belief that it is not part of their professional scope of practice. This lack of knowledge is present across the profession and having a specialized post-graduate training does not guarantee the achievement of the required competence. All the above may impact on appropriate triage and management and in some cases have the potential to negatively impact both patient and practitioner. We strongly encourage Italian public administrators and national council representatives to utilize our study as a starting point for the implementation of a stronger cardiovascular screening competencies framework within the Italian physiotherapy core curriculum.

## Supplementary Information

**Additional file 1.** Survey developed based on recent scientific publications and adapted to the Italian socio-economic and cultural context.

**Additional file 2.** Results of the non-parametric test to identify any significant difference in responses between subgroups of the sample.

**Additional file 3.** Blood pressure screening in physiotherapy practice.

**Additional file 4.** Blood pressure screening during exercise in physiotherapy practice.

**Additional file 5.** Decision tool for early identification of potential cardiovascular adverse event.

**Additional file 6. **Raw dataset.

## Data Availability

The datasets generated and/or analysed during the current study are available and provided are as a supplementary file.

## Abbreviations

CVD: Cardiovascular disease
BP: Blood pressure
HR: Heart rate
HBP: High blood pressure
SBP: Systolic blood pressure
DBP: Diastolic blood pressure
ISH: International society of Hypertension
ESH: European society of Hypertension
AHA: American Heart Association
OMPT: Orthopedic manipulative physical therapy
