# Supplementary material for: Assessing cardiovascular parameters and risk factors in physical therapy practice: findings from a cross-sectional national survey and implication for clinical practice
Source: BMC Musculoskelet Disord. 2022 Aug 4;23:749. doi: 10.1186/s12891-022-05696-w (PMC9351255; doi:10.1186/s12891-022-05696-w)
Supplement: Supplementary file 1 — Additional file 1. Survey developed based on recent scientific publications and adapted to the Italian socio-economic and cultural context. [file 12891_2022_5696_MOESM1_ESM.pdf]

QUESTIONNAIRE:

Are you a physiotherapist practising in Italy? If you are not practising in Italy please select NO, to exit the questionnaire
Yes
No

SECTION 1. DEMOGRAPHICS, PRACTICE SETTINGS AND THE EDUCATION LEVEL CHARACTERISTICS
This information will be used to compare different groups of physiotherapists

- What is your highest earned degree?
BSc
MSc
PhD
- Did you earn an IFOMPT OMPT specialization?
Yes
No
- How many years have you been practicing as a licensed physical therapist?
0-5
6-10
11-15
16-20
20+
- What physical therapy setting(s) do you currently practice in? (Check all that apply)
Private practice (primary line care)
Hospital (secondary care line)
Education
Research
- What main physical therapy access regimen do you practice in?
Direct access
Secondary care referral pathway

SECTION 2. KNOWLEDGE OF CURRENT PRACTICE RECOMMENDATIONS, CARDIOVASCULAR PARAMETERS, RISK FACTORS, BLOOD PRESSURE MEASUREMENT TECHNIQUE AND CLINICAL MANAGEMENT

KNOWLEDGE OF GUIDELINES RELEVANT TO CARDIOVASCULAR PARAMETERS ASSESSMENT
- Are you familiar with guidelines relevant to cardiovascular parameters assessment?
Yes
No
- If yes, which international guidelines are you familiar with*
NICE
American Heart Association
Italian Society of Hypertension
European Society of Cardiology and European Society of Hypertension

KNOWLEDGE OF CARDIOVASCULAR PARAMETERS ASSESSMENT
- Do you consider cardiovascular parameters assessment in your practice?
Yes
No
- If not, why you don't evaluate the cardiovascular parameters?
Not relevant for my practice
Outside the physical therapy's scope
Work in secondary care referral pathway
- If yes, what cardiovascular parameter do you evaluate? *
Heart rate
Blood pressure
Oxygen saturation
None
- If yes, what are the most relevant risk factors? *
Smoke
Diet
Level of fitness
Cholesterol
Diabetes
Obesity
Chronic Kidney disease
Familiarity
Old age
Socioeconomic level
Gender
Sleep apnea
Stress
- Which are the normal, high-normal blood pressure and hypertension ranges (systolic blood pressure; diastolic blood pressure) defined in the most recent guidelines?
Normal (SBP; DBP)
High-normal (SBP; DBP)
Hypertension (SBP; DBP)
- Which are the normative values of the heart rate defined in the most recent guidelines?
Normal
Bradycardia
Tachycardia

EDUCATION IN CARDIOVASCULAR PARAMETERS ASSESSMENT
- Have you ever received an adequate training, or have you ever done any specific courses on the measurement of the cardiovascular parameters?
Yes
No
- If yes, where did you learn these notions? *
Workplace
Continuing Professional Development courses
During the Bachelor
During the Master
Interaction with other healthcare professionals

Personal readings (scientific books or literature)
Social media and Podcast

ABOUT THE USE OF CARDIOVASCULAR PARAMETERS ASSESSMENT
- How relevant is cardiovascular parameters assessment in your practice?
Likert (0-10)
- Do you measure blood pressure and/or heart rate in your practice?
Yes
No
- If No, why? *
Outside the physical therapy scope of practice
Working in a secondary care referral pathway (patients previously evaluated by a physician)
Not trained adequately
Requires too much time

CONFIDENCE USING CARDIOVASCULAR PARAMETERS ASSESSMENT
- Quantify your ability in conducting a blood pressure assessment
Not confident
Insecure
Quite sure
Sure
- Quantify your confidence in interpreting the findings within your blood pressure assessment
Not confident
Insecure
Quite sure
Sure
- Quantify your confidence in managing the findings within your blood pressure assessment
Not confident
Insecure
Quite sure
Sure
- Quantify your ability in conducting a heart rate assessment
Not confident
Insecure
Quite sure
Sure
- Quantify your confidence in interpreting the findings within your heart rate assessment
Not confident
Insecure
Quite sure
Sure
- Quantify your confidence in managing the findings within your heart rate assessment
Not confident
Insecure
Quite sure
Sure
- How many baseline blood pressure measurements are recommended?
1
2
3
4
5
- Where should the blood pressure measurement has to be performed?
Right arm
Left arm
Both
No difference
- If both, how do you evaluate the blood pressure?
Monitor both arm
Average the measurements of the two arms
Monitor the arm with the highest blood pressure
No specific indication

- How would you manage any serious anomalies detected during the evaluation of cardiovascular parameters? *
Monitoring patient's symptoms
Refer to general practitioner
Referral to the Emergency Department
Referral to a Specialist
Request further examination

ATTITUDES TOWARDS CARDIOVASCULAR RISK
- To what extent do you consider cardiovascular risk in your practice?
Likert (0-10)
- Do you screen for cardiovascular risk in your practice?
Yes
No
- Do you evaluate your patients' cardiovascular fitness before exercises?
Yes
No
- If yes, what tools or tests do you use to assess the cardiovascular fitness of your patients? *
Maximal incremental test
6 minutes walking test
3 minutes step up test
Indirect tests
None
- Do you monitor blood pressure values during and/or post-exercise?
Yes
No
- If yes, please specify the current recommended upper blood pressure threshold (systolic blood pressure; diastolic blood pressure) triggering the cessation of exercise
Exaggerated Blood Pressure response to exercise (SBP; DBP)

SECTION 3. FORMAL EDUCATION AND PERSONAL OPINIONS

TRAINING IN CARDIOVASCULAR PARAMETERS ASSESSMENT

- How much relevant do you consider training in cardiovascular parameter assessment?

Likert (0-10)

- How much relevant do you consider training in cardiovascular risk assessment/management (e.g., syncopal events, tachycardia etc.)?

Likert (0-10)

- How training in conducting a cardiovascular parameter assessment should be provided? *

Within the under-graduate programs (Bachelor)

Within post-graduate programs (Masters)

Within Continuing Professional Development courses

In the workplace
