# Supplementary material for: Assessing cardiovascular parameters and risk factors in physical therapy practice: findings from a cross-sectional national survey and implication for clinical practice
Source: BMC Musculoskelet Disord. 2022 Aug 4;23:749. doi: 10.1186/s12891-022-05696-w (PMC9351255; doi:10.1186/s12891-022-05696-w)
Supplement: Supplementary file 2 — Additional file 2. Results of the non-parametric test to identify any significant difference in responses between subgroups of the sample. [file 12891_2022_5696_MOESM2_ESM.docx]

Additional file 2. Results of the non-parametric test to identify any significant difference in responses between subgroups of the sample; p-values are reported.

| **Question** | **Highest earned Degree** | **OMPT qualification** | **access regimen** | **years of practice** |
| --- | --- | --- | --- | --- |
| Are you familiar with guidelines relevant to cardiovascular parameters assessment? | 0.443 | 0.826 | 0.328 | 0.970 |
| Do you consider cardiovascular parameters assessment in your practice? | 0.386 | 0.729 | 0.311 | 0.053 |
| Which are the normal blood pressure ranges (systolic blood pressure; diastolic blood pressure) defined in the most recent guidelines? | 0.145 | 0.030^$^ | 0.603 | 0.344 |
| Which are the high-normal blood pressure ranges (systolic blood pressure; diastolic blood pressure) defined in the most recent guidelines? | 0.243 | 0.086 | 0.776 | 0.352 |
| Which are the hypertension ranges (systolic blood pressure; diastolic blood pressure) defined in the most recent guidelines? | 0.854 | 0.492 | 0.616 | 0.087 |
| Which are the normative values of the normal heart rate defined in the most recent guidelines? | 0.632 | 0.525 | 0.550 | 0.959 |
| Which are the normative values of tachycardia defined in the most recent guidelines? | 0.710 | 0.197 | 0.185 | 0.954 |
| Which are the normative values of Bradycardia defined in the most recent guidelines? | 0.707 | 0.230 | 0.518 | 0.736 |
| How relevant is cardiovascular parameters assessment in your practice? | 0.283* | 0.095* | 0.171* | 0.493** |
| Do you measure blood pressure and/or heart rate in your practice? | 0.789 | 0.967 | 0.944 | 0.168 |
| Quantify your ability in conducting a blood pressure assessment | 0.848 | 0.610 | 0.029^$^ | 0.027^$^ |
| Quantify your confidence in interpreting the findings within your blood pressure assessment | 0.231 | 0.150 | 0.033^$^ | 0.056 |
| Quantify your confidence in managing the findings within your blood pressure assessment | 0.648 | 0.337 | 0.208 | 0.076 |
| Quantify your ability in conducting a heart rate assessment | 0.856 | 0.517 | 0.054 | 0.396 |
| Quantify your confidence in interpreting the findings within your heart rate assessment | 0.246 | 0.102 | 0.996 | 0.837 |
| Quantify your confidence in managing the findings within your heart rate assessment | 0.534 | 0.347 | 0.646 | 0.416 |
| How many baseline blood pressure measurements are recommended? | 0.639 | 0.537 | 0.678 | 0.280 |
| Where should the blood pressure measurement has to be performed? | 0.700 | 0.429 | 0.621 | 0.439 |
| If both, how do you evaluate the blood pressure? | 0.851 | 0.130 | 0.097 | 0.023^$^ |
| To what extent do you consider cardiovascular risk in your practice? | 0.891* | 0.563* | 0.754* | 0.045** |
| Do you screen for cardiovascular risk in your practice? | 0.855 | 0.462 | 0.213 | 0.899 |
| Do you evaluate your patients’ cardiovascular fitness before exercises? | 0.706 | 0.732 | 0.948 | 0.950 |
| Do you monitor blood pressure values during and/or post-exercise? | 0.458 | 0.638 | 0.188 | 0.118 |
| How much relevant do you consider training in cardiovascular risk assessment/management (e.g., syncopal events, tachycardia etc.)? | 0.073* | 0.279* | 0.563* | 0.355** |
| How much relevant do you consider training in cardiovascular parameter assessment? | 0.630* | 0.658* | 0.759* | 0.287** |

**Note:** the reported p-value are referred to chi squared test, except * that are referred to Mann Whitney Test and ** that are referred to Kruskal-Wallis test; $ = post-hoc analyses did not reveal any significant differences between all comparisons.
