# Supplementary material for: Assessing cardiovascular parameters and risk factors in physical therapy practice: findings from a cross-sectional national survey and implication for clinical practice
Source: BMC Musculoskelet Disord. 2022 Aug 4;23:749. doi: 10.1186/s12891-022-05696-w (PMC9351255; doi:10.1186/s12891-022-05696-w)
Supplement: Supplementary file 3 — Additional file 3. Blood pressure screening in physiotherapy practice. [file 12891_2022_5696_MOESM3_ESM.pdf]

BLOOD PRESSURE SCREENING IN PHYSICAL THERAPY CLINICAL PRACTICE

Based on the article:

ASSESSING CARDIOVASCULAR PARAMETERS AND RISK FACTORS IN PHYSICAL THERAPY PRACTICE: FINDINGS FROM A CROSS-SECTIONAL NATIONAL SURVEY AND IMPLICATION FOR CLINICAL PRACTICE

A. Faletra, G. Bellin, J. Dunning, C. Fernández-de-las-Peñas, L. Pellicciari, F. Brindisino, E. Galeno, G. Rossettini, F. Maselli, R. Severin, F. Mourad

Why is it important to routinely perform a Blood Pressure screening in Physical Therapy practice?

1. Raised Blood Pressure is the leading cause of death and comorbidities globally [1]
2. Hypertension is associated with cardiac arrythmia and myocardial infarction
3. Hypertension is often asymptomatic [2]
3. 62% of patients undergoing physical therapy has history of cardiac disease
4. The risk of acute myocardial infarction during exercise is seven times higher than that of sudden cardiac death [2]

Physiotherapists could play an important role in the early recognition of cardiovascular risk factors and are therefore advised to:

1. Screen for risk factors and bad lifestyle habits during medical history
2. Assess Blood Pressure following a standardized procedure, not only at rest but also during exercise [2]
3. Monitor Blood Pressure parameters and/or act on referral during the rehabilitation journey

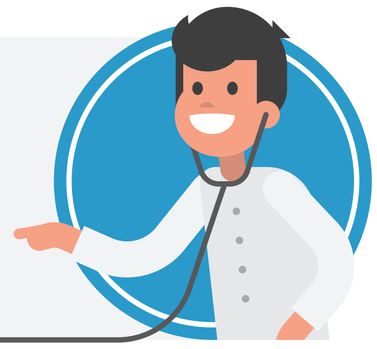

STANDARDIZED PROCEDURE FOR BLOOD PRESSURE SCREENING

INTERPRETATION
· If the blood pressure is ≥ 140-90 mmhg it is recommended to monitor over 2-3 visits or to refer to the general practitioner in order to confirm a diagnosis of Hypertension

PROTOCOL
· At each visit take 3 measurements with 1 min among them of both arms, possibly simultaneously; calculate the average of the last 2 measurements. If Blood Pressure of the first reading is < 130/85 mmHg no further measurement is required;

· If the difference between the two arms is greater than 10 mmHg, use the one with the highest pressure for monitoring.

CUFF
· Size according to the individual's arm circumference;

· For manual auscultatory devices the inflatable bladder of the cuff must cover 75%-100% of the individual's arm circumference. For electronic devices use cuff according to device instructions.

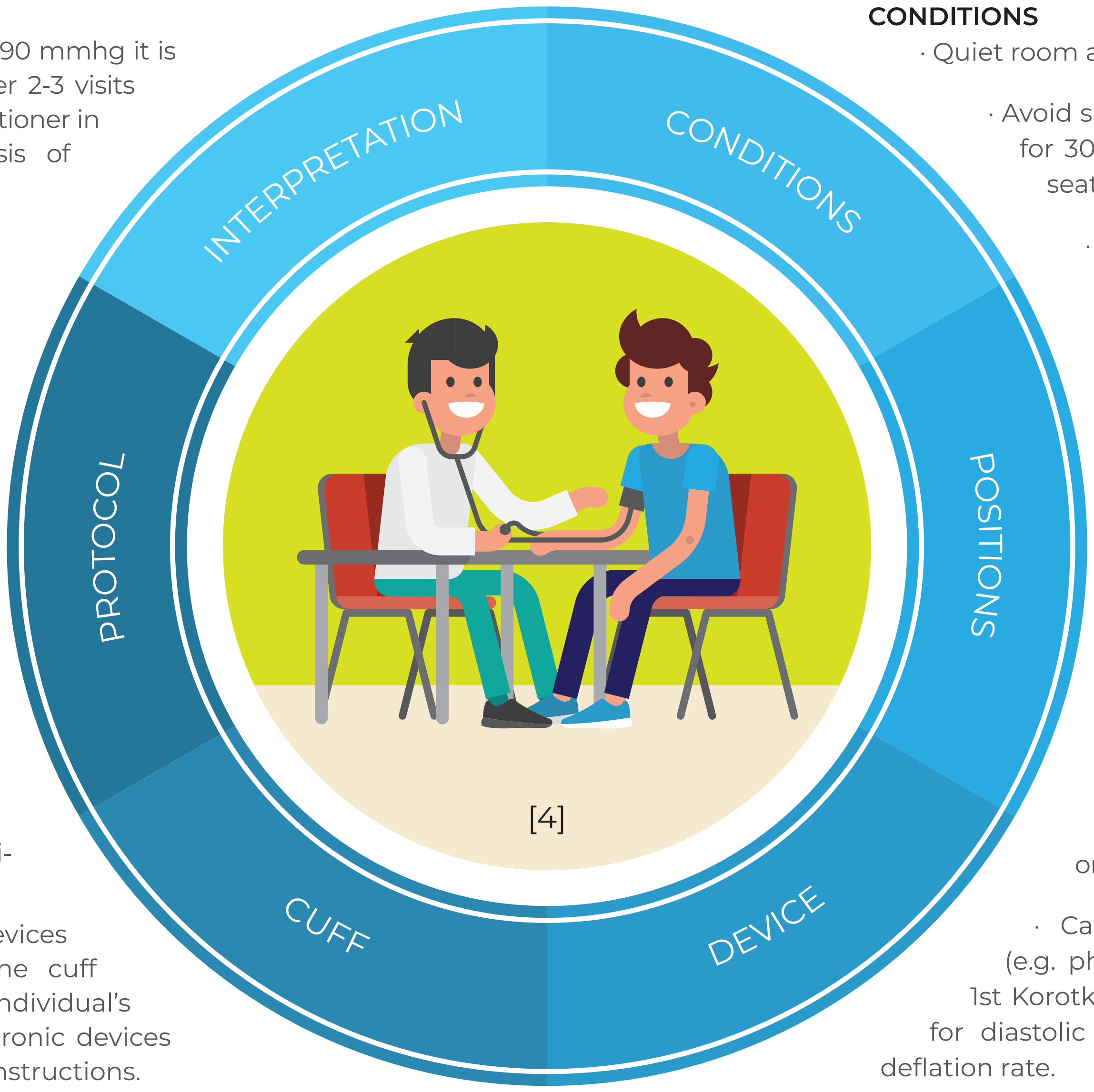

CONDITIONS
· Quiet room and comfortable temperature;

· Avoid smoking, caffeine and exercise for 30 min; empty bladder; remain seated and relaxed for 3-5 min;

· Avoid talking during procedure.

POSITIONS
· Sitting: arm resting on the table with mid-arm at heart level; back supported; leg uncrossed and feet flat on floor.

DEVICE
· Validated electronic (oscillometric) upper-arm cuff device (automated/hybrid oscillometric device).
A wide range of accurate electronic devices is available on the market;

· Calibrated auscultatory device (e.g. phonendoscope) to detect the 1st Korotkoff sound for systolic and 5th for diastolic blood pressure, with a low deflation rate.

THE FULL AND DETAILED DESCRIPTION OF THE PROTOCOL CAN BE FOUND IN THE LATEST GUIDELINES:
https://journals.lww.com/jhypertension/Fulltext/2020/06000/2020_International_Society_of_Hypertension_global.2.aspx

1. Whelton et al., 2017 ACC/AHA/AAPA/ABC/ACPM/AGS/APhA/ASH/ASPC/NMA/PCNA Guideline for the Prevention, Detection, Evaluation, and Management of High Blood Pressure in Adults: A Report of the American College of Cardiology/American Heart Association Task Force on Clinical Practice Guidelines. J Am Coll Cardiol 2018, 71(19):e127-e248.
2. Stergiou et al., 2021 European Society of Hypertension practice guidelines for office and out-of-office blood pressure measurement. J Hypertens 2021, 39(7):1293-1302.
3. Unger, T., et al., 2020 International Society of Hypertension Global Hypertension Practice Guidelines. Hypertension, 2020. 75.
4. Severin, R., et al., Blood Pressure Screening by Outpatient Physical Therapists: A Call to Action and Clinical Recommendations. Phys Ther, 2020.
