# Supplementary material for: Assessing cardiovascular parameters and risk factors in physical therapy practice: findings from a cross-sectional national survey and implication for clinical practice
Source: BMC Musculoskelet Disord. 2022 Aug 4;23:749. doi: 10.1186/s12891-022-05696-w (PMC9351255; doi:10.1186/s12891-022-05696-w)
Supplement: Supplementary file 4 — Additional file 4. Blood pressure screening during exercise in physiotherapy practice. [file 12891_2022_5696_MOESM4_ESM.pdf]

BLOOD PRESSURE SCREENING DURING EXERCISE

Based on the article:

ASSESSING CARDIOVASCULAR PARAMETERS AND RISK FACTORS IN PHYSICAL THERAPY PRACTICE: FINDINGS FROM A CROSS-SECTIONAL NATIONAL SURVEY AND IMPLICATION FOR CLINICAL PRACTICE

A. Faletra, G. Bellin, J. Dunning, C. Fernández-de-las-Peñas, L. Pellicciari, F. Brindisino, E. Galeno, G. Rossettini, F. Maselli, R. Severin, F. Mourad

Why should physiotherapists routinely perform a Blood Pressure screening on their patients in preparation for or during exercises?

1. Masked hypertension (normal blood pressure at office, but elevated outside the office environment) is an important clinical risk factor related to cardiovascular disease [1]

2. Masked hypertension is associated with obesity, old age, smoking, alcohol consumption, sedentary lifestyle, use of contraceptives [2]

3. An excessive increase of blood pressure during submaximal exercise is a risk factor associated with future hypertension, stroke, congestive heart failure, ischemic heart disease and cardiovascular mortality [3]

4. Central Blood Pressure (pressure in the ascending aorta, just outside left ventricle) is also affected during strengthening exercises [4]

5. The risk of acute myocardial infarction during exercise is seven times higher than that of sudden cardiac death [5]

Physiotherapists, in order to reduce the likelihood of adverse cardiac events during exercise, should:

1. Screen Blood Pressure in preparation, during and post exercise with a standardized procedure;
2. Avoid any physical exercise prescription if Systolic Blood Pressure or Diastolic Blood Pressure > 200/100 mmHg;
3. Screen of blood pressure must correlate to medical history and other comorbidities;
4. Refer for medical consultation in case of excessive levels of Blood Pressure (too high) during sub-maximal exercise;
5. Monitor Blood Pressure before, during and after strengthening exercise. Due to the lack of specific recommendations for screening during strengthening exercise it is advised to follow the recent guidelines for aerobic exercise [6]

SCREENING OF BLOOD PRESSURE DURING SUB-MAXIMAL EXERCISE

CUT-OFF FOR MEDICAL REFERRAL
· Maximal exercise Systolic Blood Pressure ≥ 210 mmHg (men) or ≥ 190 mmHg (women)

· Maximal exercise Diastolic Blood Pressure ≥ 110 mmHg (men and women)

AFTER EXERCISE
· Measure Blood Pressure immediately post-exercise every 2 min for 6-8 min or longer if Blood Pressure is not returned to near baseline levels

· Organize follow-up testing for subject with abnormal exercise induced BP responses

DURING EXERCISE
· Measure Blood Pressure each 2-3 min or more frequently in high risk subjects

· For manual measurement inflate to 30 mmHg above Systolic Blood Pressure;

· For manual measurement use Korakoff sound 1st and 5th for Systolic Blood Pressure and Diastolic Blood Pressure, respectively

· Technical difficulties with inability to measure Systolic Blood Pressure is an absolute indication for test termination.

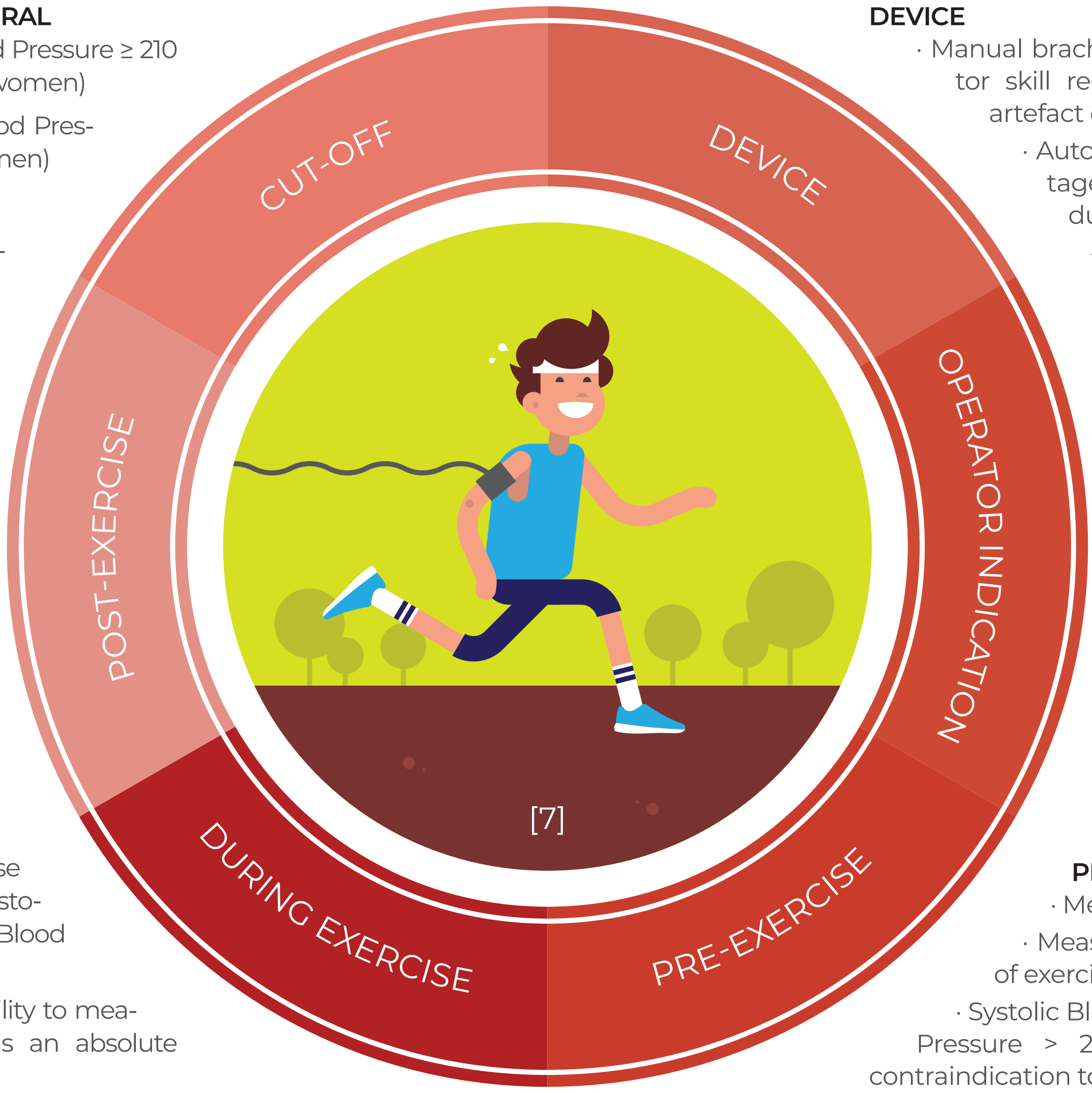

DEVICE
· Manual brachial cuff (disadvantages: operator skill required, movement and noise artefact disrupt accuracy);

· Automated brachial cuff (disadvantages: expensive, wrong reading due to artefacts);

· Finger cuff (disadvantages: overestimate brachial Systolic Blood Pressure);

· Periodic calibration is needed.

OPERATOR
· Use a correct size cuff;

· For manual measurement, avoid excess stethoscope pressure;

· Measure Blood Pressure each time with the cuff supported at heart level, shoulder/arm relaxed and no talking;

· Subject to avoid gripping on the treadmill rails.

PRE-EXERCISE
· Measure supine Blood Pressure;

· Measure Blood Pressure in posture of exercise;

· Systolic Blood Pressure or Diastolic Blood Pressure > 200/100 mmHg is a relative contraindication to exercise testing.

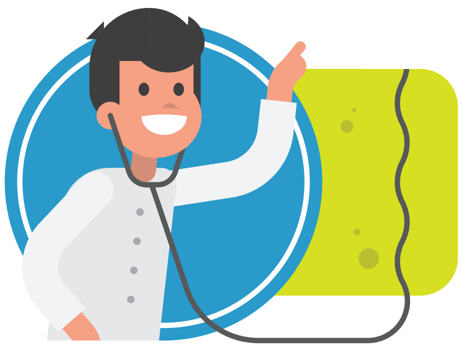

1. Peacock, J., et al., Unmasking masked hypertension: prevalence, clinical implications, diagnosis, correlates and future directions. J Hum Hypertens, 2014. 28(9): p. 521-8.
2. Kayrak, M., et al., Exaggerated blood pressure response to exercise--a new portent of masked hypertension. Clin Exp Hypertens, 2010. 32(8): p. 560-8.
3. Schultz, M.G., et al., Masked hypertension is "unmasked" by low-intensity exercise blood pressure. Blood Press, 2011. 20(5): p. 284-9.
4. Tanaka, S., et al., Differential response of central blood pressure to isometric and isotonic exercises. Sci Rep, 2014. 4: p. 5439.
5. Risgaard, B., et al., Sports-related sudden cardiac death in a competitive and a noncompetitive athlete population aged 12 to 49 years data from an unselected nationwide study in Denmark. Heart Rhythm, 2014. 11(10): p. 1673-81.
6. Unger, T., et al., 2020 International Society of Hypertension Global Hypertension Practice Guidelines. Hypertension, 2020. 75(6): p. 1334-1357.
7. Sharman, J.E. and A. LaGerche, Exercise blood pressure: clinical relevance and correct measurement. J Hum Hypertens, 2015. 29(6): p. 351-8.
8. Currie et al., Exercise Blood Pressure Guidelines: Time to Re-evaluate What is Normal and Exaggerated? Sports Med 2018, 48(8):1763-1771.
9. Severin, R., et al., Blood Pressure Screening by Outpatient Physical Therapists: A Call to Action and Clinical Recommendations. Phys Ther, 2020.
