# Supplementary material for: Assessing cardiovascular parameters and risk factors in physical therapy practice: findings from a cross-sectional national survey and implication for clinical practice
Source: BMC Musculoskelet Disord. 2022 Aug 4;23:749. doi: 10.1186/s12891-022-05696-w (PMC9351255; doi:10.1186/s12891-022-05696-w)
Supplement: Supplementary file 5 — Additional file 5. Decision tool for early identification of potential cardiovascular adverse event. [file 12891_2022_5696_MOESM5_ESM.pdf]

DECISION TOOL FOR EARLY IDENTIFICATION OF POTENTIAL CARDIOVASCULAR ADVERSE EVENTS

Decide your clinical action based on the level of concern

Based on the article:

ASSESSING CARDIOVASCULAR PARAMETERS AND RISK FACTORS IN PHYSICAL THERAPY PRACTICE: FINDINGS FROM A CROSS-SECTIONAL NATIONAL SURVEY AND IMPLICATION FOR CLINICAL PRACTICE

A. Faletra, G. Bellin, J. Dunning, C. Fernández-de-las-Peñas, L. Pellicciari, F. Brindisino, E. Galeno, G. Rossettini, F. Maselli, R. Severin, F. Mourad

LEVEL OF CONCERN

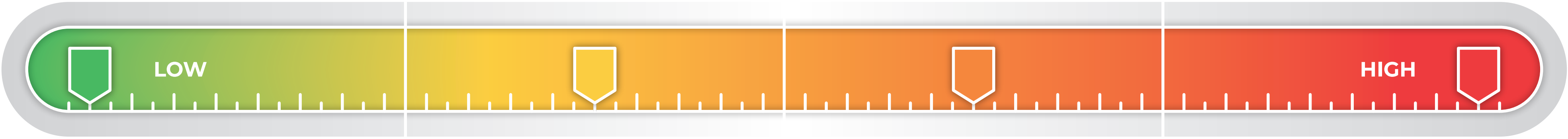

NO CONCERNING FEATURES

Revise management if clinical features change unexpectedly. Also, attention must be paid for 'masked hypertension' and then a blood pressure assessment during exercises is advised for those subjects presenting cardiovascular features at the history taking. These patients are at high risk of fatal adverse events as the blood pressure sharply increase during low-intensity physical exertions.

LOW RISK PROFILE FEW CONCERNING

Treat & monitor progress closely (vigilance). However, a further blood pressure assessment during exercises or a medical consultation is advised before low-intensity exercises.

MODERATE RISK PROFILE SOME CONCERNING FEATURES

DO NOT treat
URGENT referral in warranted

HIGH RISK PROFILE CONCERNING FEATURES

DO NOT treat
EMERGENCY referral is warranted

NO CONCERNING FEATURES	LOW RISK PROFILE FEW CONCERNING	MODERATE RISK PROFILE SOME CONCERNING FEATURES	HIGH RISK PROFILE CONCERNING FEATURES
Normal Blood Pressure & no other Risk Factors.	High-normal Blood Pressure & no other Risk Factors or 1/2 Risk Factors	High-normal Blood Pressure & ≥ 3 Risk Factors	High-normal Blood Pressure & HMOD, Chronic Kidney Disease Grade 3, CVD
	Grade 1 hypertension & no other Risk Factors	Grade 1 hypertension & 1/2 Risk Factors	Grade 1 hypertension & ≥ 3 Risk Factors or Chronic Kidney Disease Grade 3 or CVD
		Grade 2 hypertension & no other Risk Factors	Grade 2 hypertension & no other Risk Factors
			Grade 2 hypertension & >2 Risk Factors or Chronic Kidney Disease Grade 3 or CVD

LEVEL OF BLOOD PRESSURE	SYSTOLIC BLOOD PRESSURE (mmHg)		DIASTOLIC BLOOD PRESSURE (mmHg)
NORMAL	<130	and	<85
NORMAL HIGH BLOOD PRESSURE	130-139	and/or	85-89
GRADE 1 HYPERTENSION	140-159	and/or	90-99
GRADE 2 HYPERTENSION	>160	and/or	>100

RISK FACTORS: Age (>65 years), sex (male>female), heart rate (>80 beats/min), increased body weight, diabetes, High LDL-C/triglyceride, family history of CVD, family history of hypertension, early-onset menopause, smoking habits, psychosocial or socioeconomic Factors, HMOD, previous coronary heart disease (CHD), HF, stroke, peripheral vascular disease, atrial fibrillation, CKD stage 3+.

HMOD: hypertension-mediate organ damage (brain, heart, kidney, central and peripheral arteries, eyes)
HF: Heart Failure
CKD: Chronic Kidney Disease
CVD: CardioVascular Disease

REFERENCES

1. Severin et al., Blood Pressure Screening by Outpatient Physical Therapists: A Call to Action and Clinical Recommendations. Phys Ther. 2020 Jun 23;100(6):1008-1019.
2. Unger et al., 2020 International Society of Hypertension Global Hypertension Practice Guidelines. Hypertension. 2020 Jun;75(6):1334-1357.
3. Whelton et al., 2017 ACC/AHA/AAPA/ABC/ACPM/AGS/APhA/ASH/ASPC/NMA/PCNA Guideline for the Prevention, Detection, Evaluation, and Management of High Blood Pressure in Adults: A Report of the American College of Cardiology/American Heart Association Task Force on Clinical Practice Guidelines. Hypertension. 2018 Jun;71(6):e13-e115.
